# Narrow complex tachycardia with alternating R-R intervals during physical stress: Double ventricular excitation

**Published:** 2008-04-01

**Authors:** Roman Laszlo, Hans-Joerg Weig, Slawomir Weretka, Juergen Schreieck

**Affiliations:** Department of cardiology, University of Tuebingen, Germany

**Keywords:** Double ventricular excitation, dual atrioventricular node conduction physiology

## Case presentation

A 44 year old man presented with recurrent symptomatic palpitations,  always appearing during physical stress each with a similiar extent and never during resting periods. He also had fatigue and moderate dyspnoea during the episodes. The past medical history of the patient did not reveal any significant diseases. Findings of the attending physician including 12 lead ECG and echocardiography did not show any abnormality. As there was no electrocardiographic documentation of the episode, a 12-lead Holter evaluation was done.

**Figure 1:**
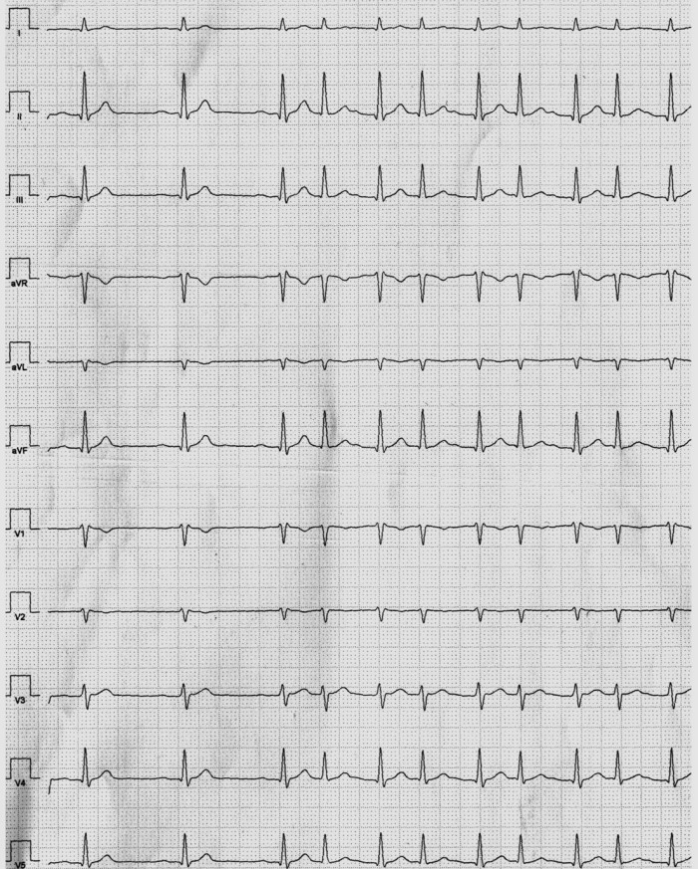
Corresponding extract of 12 lead Holter ECG when our patient felt typical palpitations.

When the patient returned one week later, he reported of a typical episode of palpitations. The corresponding extract of the Holter ECG is shown in Figure 1.

In synopsis of the findings we saw the indication for an invasive electrophysiology study due to a documented paroxysmal narrow complex tachycardia with alternating R-R intervals coming along with symptomatic palpitations.

An octapolar catheter was placed in the coronary sinus with distal and proximal pair of electrodes configured as CS 1-2 and CS 7-8 respectively. Dual atrioventricular conduction properties of the AV node were verified with programmed atrial stimulation with premature coupling of one extrastimulus (« 500 S3 ») showing a typical AH-jump. No retrograde ventriculoatrial conduction was observed in our patient.

**Figure 2:**
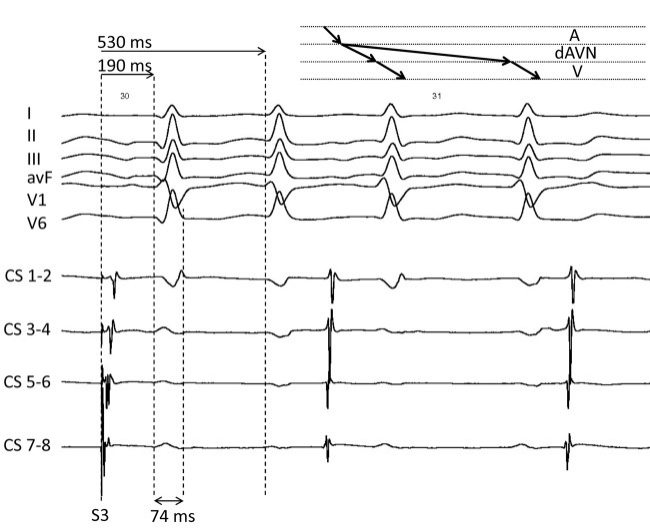
Onset of a narrow complex tachycardia induced by programmed atrial stimulation with premature coupling of one extrastimulis (« 400 S3 ») during orciprenalin infusion. I, II, III, avF, V1, V6 = surface ecg ; CS 1-2, CS 3-4, CS 5-6, CS 7-8 = distal bipole, bipole 3-4, bipole 5-6, proximal bipole of coronary sinus catheter respectively ; A= atrium, dAVN= AV node with dual conduction properties, V= ventricle.

Under basal conditions no tachycardia was inducable, so we decided to use an orciprenaline infusion to simulate physical strain as the tachycardia of our patient always appeared under these conditions. Finally, during orciprenaline infusion and atrial pacing via CS 7-8 with premature coupling of one extrastimulus (« 400 S3 »), a narrow complex tachycardia was induced (onset see Figure 2). During tachycardia our patient felt his typical symptoms. What is the mechanism ?

## Discussion

**Figure 3:**
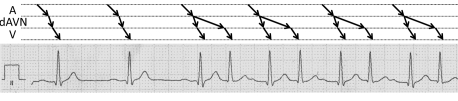
Same extract of the 12 lead Holter ECG as in Figure 1. The ladder diagram indicates that 1 : 2 atrioventricular conduction in the presence of dual atrioventricular nodal pathways also fits as a pathomechansim for the initially documented tachycardia. A= atrium, dAVN= AV node with dual conduction properties, V= ventricle.

The tachycardia of our patient was caused by a frequency-dependent 1 : 2 atrioventricular conduction in presence of dual atrioventricular nodal pathways. In retrospect, this pathomechanism also fits to the tachycarida initially documented in the Holter ECG recordings, as shown in Figure 3.

1 : 2 atrioventricular conduction (= double ventricular response to a single sinus or atrial impulse resulting in two QRS complexes for one P wave) is a rare manifestation of dual atrioventricular nodal pathways. Two electrophysiological characteristics are basic requirements for a double ventricular depolarisation due to a dual AV node and simultaneous absence of the inducibility of a typical AV-nodal reentry tachycardia: [1] 1) Absence of retrograde ventriculoatrial conduction via the fast as well as the slow pathway 2) Time difference between antegrade conduction via slow pathway and antegrade conduction time via fast pathway has to be longer than the effective refractory period of the infranodal conduction system. Slow pathway modifcation is considered to be a curative therapeutical approach for this rare form of narrow complex tachycardia.

In conclusion, we have induced the clinical tachycardia of our patient. The patient underwent successful slow pathway modification with complete disapperance of symptoms and electrocardiographic manifestations of 1 : 2 atrioventricular conduction.
